# Relating Specific Emotions to Intrinsic Motivation: On the Moderating Role of Positive and Negative Emotion Differentiation

**DOI:** 10.1371/journal.pone.0115396

**Published:** 2014-12-17

**Authors:** Leen Vandercammen, Joeri Hofmans, Peter Theuns

**Affiliations:** Department of Psychology, Vrije Universiteit Brussel, Elsene, Belgium; Southwest University, China

## Abstract

Despite the fact that studies on self-determination theory have traditionally disregarded the explicit role of emotions in the motivation eliciting process, research attention for the affective antecedents of motivation is growing. We add to this emerging research field by testing the moderating role of emotion differentiation –individual differences in the extent to which people can differentiate between specific emotions– on the relationship between twelve specific emotions and intrinsic motivation. To this end, we conducted a daily diary study (*N* = 72) and an experience sampling study (*N* = 34). Results showed that the relationship between enthusiasm, cheerfulness, optimism, contentedness, gloominess, miserableness, uneasiness (in both studies 1 and 2), calmness, relaxation, tenseness, depression, worry (only in Study 1) on one hand and intrinsic motivation on the other hand was moderated by positive emotion differentiation for the positive emotions and by negative emotion differentiation for the negative emotions. Altogether, these findings suggest that for people who are unable to distinguish between different specific positive emotions the relationship between those specific positive emotions and intrinsic motivation is stronger, whereas the relationship between specific negative emotions and intrinsic motivation is weaker for people who are able to distinguish between the different specific negative emotions. Theoretical and practical implications are discussed.

## Introduction

Motivation is central to the functioning of organizations and their employees. Motivated employees perform well, are more satisfied and committed to their jobs, and are less absent [1]–[4]. Because of the central role of motivation for both the individual and the organization, a thorough understanding of the mechanisms underlying it is crucial, both from a theoretical and a practical point of view. Throughout the years, this awareness has led to the development of a series of motivation theories such as equity theory [5], expectancy theory [6], and incentive theory [7]. Among this multitude of motivation theories, the most popular and comprehensive one at present is without doubt self-determination theory (SDT). SDT combines insights of several of the more traditional motivation theories, and is empirically supported in numerous domains such as education [8], sports [9], psychotherapy [10], health care [11], and work and organizational psychology [12]–[14].

One of the core ideas of SDT is that motivation is elicited by the satisfaction of three basic psychological needs (i.e., the need for autonomy, competence, and relatedness). As a result, research on SDT has traditionally focused on the predictive role of satisfaction of these three basic psychological needs for motivation. Recently, however, there is growing awareness that motivation (in SDT) is not only triggered by general need satisfaction, but also by more specific affective experiences [15], [16].

Despite recent support for the key role of emotions in the motivation-generative process of SDT, research on the boundary conditions of this relationship is missing. This is an important limitation as such research would allow the identification of conditions under which emotions relate to motivation, thereby leading to a better understanding of the mechanisms underlying the relationship between emotions and motivation. To address this limitation, we introduce the concept of emotion differentiation, or the extent to which people experience their emotions as a single affective state (i.e., low emotion differentiation) rather than specific, well-separated affective states (i.e., high emotion differentiation). Because individual differences in emotion differentiation not only affect the affective experiences themselves [17], [18], but also the extent to which these affective experiences drive subsequent behaviors [19]–[21], we expect emotion differentiation to moderate the relationship between the different specific emotions and intrinsic motivation.

In what follows, we first elaborate on SDT, then we discuss the role of emotions within SDT; subsequently, we define the concept of emotion differentiation; and finally, we explain why emotion differentiation is hypothesized to moderate the relationship between emotions and intrinsic motivation.

### Self-determination theory

According to SDT, motivation can be subdivided in two major categories, namely controlled and autonomous motivation. When employees are autonomously motivated, they experience a sense of volition and participate in activities that are in line with their own interests and values. In SDT, this type of motivation is further divided into identified and intrinsic motivation; employees who experience *identified motivation* engage in activities because these activities fit with their personal goals, whereas employees who are *intrinsically motivated* do things because they find them enjoyable and interesting as such. Controlled motivation, in turn, refers to performing activities because of internal or external pressure [22]. Two subtypes can further be distinguished, namely external and introjected motivation. When employees perform certain behaviors to avoid punishment or to receive a reward, they are *externally motivated*, whereas *introjected motivated* employees engage in activities to avoid feelings of shame and guilt [23].

According to SDT, intrinsic motivation should be considered the superior type of motivation, and in line with this claim, research has shown that it relates to positive work outcomes such as increased vitality and well-being [24], cognitive engagement [25], effective performance [26], work effort [27], and knowledge sharing [28]. Moreover, previous research has demonstrated that emotions are of key importance in the elicitation of intrinsic motivation as it refers to *“the engagement in an activity for its own sake, that is, for the satisfaction and enjoyment experienced during the course of the activity itself”* [23]. Because of these reasons, we will focus on intrinsic motivation in the present paper.

### The role of emotions within SDT

According to SDT, motivation results from the degree to which the person perceives the fulfillment (or thwarting) of three basic psychological needs: the need for autonomy, competence, and relatedness [29]. To satisfy their *need for autonomy* employees must be able to make their own choices and behave according to their own interests and values. The *need for competence* will be satisfied when employees feel capable and effective. Finally, employees who feel accepted by their colleagues and feel connected to them will have their *need for relatedness* satisfied. Intrinsic motivation is then the result of the fulfillment of those three needs, while thwarting of these needs would result in controlled motivation [8], [9], [22].

Although SDT acknowledges that the three basic psychological needs contain both a cognitive and an affective component [29], [30], there are only few SDT studies that have focused on the explicit role of emotions in the motivation-generative mechanism. This treatment of emotions is surprising, as research in the emotion domain has convincingly shown that emotions and motivation are inextricably linked [31]. In particular, according to the componential approach to emotions, an emotion consists of different emotion components, with one of them –the action readiness component– specifically referring to the readiness or unreadiness to interact with the environment. Indeed, the componential approach to emotions states that, when people encounter a certain situation, they evaluate whether the situation is relevant or not for their own well-being (i.e., does it harm or favor the individual's concerns?), and when deemed relevant, the action readiness component –together with the other components– is activated to deal with this situation [32], [33]. From this conceptualization of emotions, it is clear that emotions are directly related to motivation, and in line with this, studies have shown that emotions play an important role in the elicitation of motivation in general [34]–[38], and intrinsic motivation in particular [15], [16]. For example, it has been demonstrated that people who experience positive emotions are more motivated for a pleasant task [15], experience more interest and enjoyment while carrying out the task [39]–[41], and continue to work longer on less pleasant, and even uninteresting tasks [15]. Moreover, Vandercammen, Hofmans, and Theuns [16] found that emotions partially mediated the relationship between need satisfaction and intrinsic motivation, thereby explicitly demonstrating that emotions are important in the elicitation of intrinsic motivation. However, whereas previous research has demonstrated that emotions play a key role in the motivation-generative process [15], [16], –to our knowledge– there has been virtually no research on the boundary conditions of this relationship. This is an important issue as research on the boundary conditions of the emotion-motivation relationship would allow the identification of the circumstances under which emotions relate to motivation, thereby leading to a better understanding of the mechanisms underlying this relationship. In the present paper, we will address this issue by studying how individual differences in the extent to which people are able to differentiate between different specific emotions –a concept that is referred to as emotion differentiation [17], [18] – affect the emotion-motivation relationship.

### Emotion differentiation

Emotion differentiation pertains to the extent to which people parse their emotional experiences in a differentiated fashion [42]. People low in emotion differentiation (hereafter called poor differentiators) have difficulties disentangling different emotions of the same valence and therefore have the tendency to distinguish emotions based on the fact that they are pleasant or unpleasant [17], [18]. In other words, these people treat a range of like-valence terms interchangeably [42]. Conversely, people high in emotion differentiation (hereafter called good differentiators) experience emotions in a differentiated manner and are therefore capable of clearly distinguishing different emotions of the same valence. For example, whereas poor differentiators have difficulties separating anger and sadness, this is not a problem for good differentiators [17], [18]. Also, while poor differentiators use general positive-negative terms to express their feelings, good differentiators can express their emotions in a precise way [18].

In the present paper, we study the impact of emotion differentiation on the relationship between six positive and six negative specific emotions and intrinsic motivation. As it has been shown that emotion differentiation for positive and negative emotions are not necessarily strongly related within the same person [43], we will distinguish between positive and negative emotion differentiation in the remainder of the paper. By doing so, we want to contribute to a better understanding of the emotion-intrinsic motivation relationship.

### Emotion differentiation as a moderator of the relationship between specific emotions and intrinsic motivation

We hypothesize that the relationship between different specific negative emotions and intrinsic motivation will be weaker for people who are high on negative emotion differentiation (hereafter called good negative differentiators) than for people who are low on negative emotion differentiation (hereafter called poor negative differentiators) (Hypothesis 1). A first reason is that it has been found that good negative differentiators manage their emotions –and especially the negative ones– better [42]. As a consequence, these people are better in down-regulating the negative influence of their negative emotions on intrinsic motivation. Second, good negative differentiators have higher levels of self-awareness so that they are better at distinguishing the causes and effects of their current emotions [18], [44]–[46]. This understanding of their emotions –in combination with good emotion-regulation capacities– should put good negative differentiators in a position in which they are less (negatively) influenced by their negative emotions as they can better control these emotions and their consequences. Third, poor negative differentiators traditionally experience a mixture of negative emotions when one particular negative emotion is triggered. For example, when people are alarmed by some negative event, they typically start to worry [47]. Whereas good negative differentiators will only experience worry, poor negative differentiators will mix up this particular emotion (worry) with other negative emotions like tension, uneasiness, gloominess, depression, and miserableness. Because poor negative differentiators experience a mixture of negative emotions in such situations, we expect their overall negative experience to be stronger, thereby intensifying the negative relation between the negative emotion and intrinsic motivation. In line with this explanation, Demiralp and colleagues [43] found that, compared to healthy individuals, depressed people have more difficulties to differentiate negative emotions. When such people encounter a situation that triggers the experience of a particular negative emotion, they immediately get into a general negative mood. Consequently, they experience a whole set of negative emotions at the same time, thereby intensifying their (negative) emotional experience.

For positive emotion differentiation, we expect a similar mechanism to hold true in the sense that we hypothesize that people low on positive emotion differentiation show larger increases in intrinsic motivation in response to the experience of specific positive emotions than people high on positive emotion differentiation. The reason is that people low on positive emotion differentiation not only have increased levels of positive emotional reactivity [48], but that they also react more strongly to the positive emotions they experience [21]. In particular, Selby et al. [21] showed that for people low in positive emotion differentiation, momentary positive emotions predicted more weight loss behaviors in anorectic people than for people high in positive emotion differentiation. One reason for this might be that people low in positive emotion differentiation (poor positive differentiators) tend to experience a mixture of positive emotions when one particular positive emotion is triggered, whereas people high in positive emotion differentiation (good positive differentiators) experience only that specific positive emotion. For example, when enthusiasm is triggered in poor positive differentiators, they do not only experience enthusiasm, but rather a global positive feeling resulting from a mix of different positive emotions. As such a mix of different positive emotions consists a stronger positive emotional experience, we expect the relationship between the specific positive emotions and intrinsic motivation to be stronger for poor positive differentiators (Hypothesis 2).

Note that we argue that being a poor differentiator might be helpful when it pertains to positive emotions, while it is dysfunctional when it concerns negative emotions. Although this idea is in line with the claim of Tugade, Feldman Barrett, and Gross [49] that a lack of emotion differentiation is not always negative, research on the positive consequences of poor emotion differentiation is very scarce. This can be seen in previous studies that have shown that poor differentiators are worse at coping [49], in detecting affective signals in others [50], and in regulating their emotions [42]. One reason for this almost exclusive focus on the downsides of poor emotion differentiation is that nearly all studies have focused on negative emotion differentiation. The present study thus contributes to the literature on emotion differentiation by studying both negative and positive emotion differentiation.

The specific emotions that were included in our study were chosen based on the circumplex model of emotions [51]. This model categorizes emotions according to two orthogonal axes: valence and arousal. The valence axis categorizes emotions based on the pleasantness/unpleasantness dimension, whereas the arousal axis categorizes emotions based on the amount of arousal or activation [51], [52]. These axes are important for emotion differentiation as it has been demonstrated that people who focus more on the arousal axis are better differentiators than people who focus on the valence axis [53], [54]. To cover all quadrants of the circumplex model, we selected three emotions per quadrant that are highly relevant in a work context [55]: optimism, cheerfulness, and enthusiasm for high valence and high arousal; relaxation, contentment, and calmness for high valence and low arousal; gloominess, miserableness, and depression for low valence and low arousal, and worry, uneasiness, and tension for low valence and high arousal [51]. Note that in this context depression refers to an emotion (i.e., a momentary and dynamic state).

Because emotions and motivation are dynamic concepts that vary strongly within the individual [56]–[58], and because our theorizing regarding the effect of emotions on motivation takes place at the within-person level, we chose to study the within-person relationship between emotions and motivation. To this end, we examined the moderating role of emotion differentiation (a stable characteristic) on the relationship between within-person variation in emotions and within-person variation in motivation by means of a daily diary study and an experience sampling study. The specific combination of these two studies, each with a different design, allows cross-validating our findings.

## Study 1

In a first study, we tested the moderating effect of positive and negative emotion differentiation by means of a daily diary study in which employees were asked to report on their emotions and the level of intrinsic motivation experienced during a task they performed that day. The data of this study were also used in another publication on the mediating role of positive and negative effect in the relationship between need satisfaction and intrinsic motivation [16].

### Method

#### Participants

Seventy-one Belgian employees and one trainee from 22 different companies, 38 men and 34 women, consented to take part in the study. The mean age of the participants was 37.06 years (*SD* = 10.79). Most participants were fulltime employed (97.2%).

#### Procedure

Before the start of the diary study, participants signed an informed consent and filled in some demographic questions. At the end of each of 10 consecutive working days they received an email with a link to an online survey. First, participants were required to recall one particular task they had performed during that same working day. This task could be any task of their own choice. Next, participants were required to answer questions pertaining to their basic psychological needs, motivation, and emotions with respect to this task. To facilitate recall, participants first had to describe the task in a few keywords. Participants, for example, reported doing data analysis, following or leading a meeting, or making a presentation. In total, 522 responses were collected, which corresponds to a response rate of 72.29%. On average, each participant filled out 7.15 questionnaires. Note that we did not ask permission for this study to a review board as no intrusive questions were asked. We only asked employees to report their feelings and motivation.

#### Measurements

All questions were selected from valid and reliable questionnaires. We changed the questions to measure experiences referring to the selected task. All items had a six-points rating scale from *totally disagree* (1) to *totally agree* (6). In the present paper we focus only on emotions and intrinsic motivation.

Emotions were assessed with the QEEW [55], which measures the four quadrants of the affective circumplex model [52]. A sample item of this questionnaire is: “I felt tense during this task”.

Intrinsic motivation was assessed with the two items from the interest/enjoyment subscale of the intrinsic motivation inventory [60] with the highest factor loadings (for a similar procedure, see [16]). These items measure two central aspects of intrinsic motivation, namely interest and enjoyment [22]. These items were “I found my task very interesting” and "I would describe the task as very enjoyable". We used only two items of the intrinsic motivation inventory to reduce the response burden on the participants (see [61], [62]).

#### Analysis

An index of emotion differentiation was obtained for each individual by computing the intra-class correlation coefficient (ICC) between the emotion ratings of that individual across the different emotion assessments. Because the ICC reflects *“the agreement among self-reported emotional states for each measurement moment over time”* [49], it is particularly useful for measuring emotion differentiation. Because we distinguished between positive and negative emotion differentiation, two ICCs were computed for each individual; one for the positive and one for the negative emotions. High ICCs are then indicative of poor emotion differentiation, and low ICCs of good emotion differentiation. Note that measuring emotion differentiation by means of ICCs is common practice (see also [49], [63], [64]) because, compared to self-reports, this approach is less susceptible to social desirability biases and lack of self-insight about emotional intelligence [65].

In the next step, the moderating effect of emotion differentiation was tested. To this end, we first tested a series of two-level regression models in which intrinsic motivation was each time predicted from another specific emotion. Subsequently, we tested whether the effect of the specific emotions on intrinsic motivation varied across individuals (i.e., whether the slope was fixed or random). This was done by testing the same model with and without a random slope, and by comparing both models using a log-likelihood difference test. For reasons of parsimony, non-significant random slopes (*p*>.05) were trimmed. As a final step, we tested a series of two-level regression models in which intrinsic motivation was predicted by a specific emotion, emotion differentiation, and the cross-level interaction between the specific emotion and emotion differentiation. To save space, only the fixed effects are reported. All level-1 predictors (i.e., the different emotions) were group-mean centered (or person-centered), while the level-2 predictors (i.e., the ICC′s) were grand-mean centered. By group-mean centering the emotion scores, we removed all between-person variation from the data, thereby allowing for an analysis of true within-person relationships. All analyses were performed using version 1.1–6 of the lme4 package in R.

### Results

#### Descriptive Statistics

The means, standard deviations, and correlations of the grand-mean centered study variables are reported in Table 1 below the diagonal. Correlations between level-1 variables (i.e., emotions and intrinsic motivation) were computed on group-mean centered data, which implies that they reflect within-person associations. Correlations that involved level-2 variables (i.e., emotion differentiation) were computed on data that were aggregated data to the person-level. As such, these correlations reflect between-person differences. The correlations show that all positive (respectively negative) emotions were positively correlated (e.g., enthusiastic-cheerful: *r* = .79, *p*<.01; depressed-miserable: *r* = .62, *p*<.01), while the positive and negative emotions related to each other in a negative way (e.g., calm-tense: *r* = −.66, *p*<.01). Intrinsic motivation was positively correlated to all positive emotions (e.g., cheerful-intrinsic motivation: *r* = .38, *p*<.01) and negatively to all negative emotions (e.g., uneasy-intrinsic motivation: *r* = −.31, *p*<.01).

**Table 1:** Means, standard deviations and correlations (i.e., Correlations between level-1 variables (i.e., emotions and intrinsic motivation) were computed on group-mean centered data, while correlations that involved level-2 variables (i.e., emotion differentiation), were computed on data that were aggregated to the person-level).

	Mean (SD) study 1	Mean (SD) study 2	1	2	3	4	5	6	7	8	9	10	11	12	13	14	15
1	3.50 (1.13)	3.71 (1.27)	-	.68**	.44**	.30**	.47**	.46**	−.38**	−.32**	−.35**	−.29**	−.38**	−.42**	.09	.34	.24**
2	3.69 (1.15)	4.16 (1.42)	.79**	-	.42**	.31**	.44**	.46**	−.42**	−.33**	−.50**	−.32**	−.51**	−.55**	.19	.30	.26**
3	3.98 (1.01)	4.62 (1.31)	.57**	.53**	-	.35**	.37**	.30**	−.50**	−.36**	−.33**	−.29**	−.43**	−.47**	−.07	−.12	.10
4	4.13 (.98)	4.98 (1.25)	.43**	.44**	.44**	-	.53**	.53**	−.42**	−.34**	−.32**	−.29**	−.36**	−.42**	.27	.11	.14
5	3.99 (1.02)	4.84 (1.31)	.37**	.36**	.36**	.63**	-	.68**	−.59**	−.29**	−.28**	−.58**	−.33**	−.48**	.23	−.13	.18*
6	3.76 (1.08)	4.42 (1.40)	.47**	.45**	.44**	.65**	.64**	-	−.45**	−.27**	−.34**	−.48**	−.38**	−.43**	.27	−.05	.22**
7	2.70 (1.29)	2.68 (1.48)	−.39**	−.38**	−.32**	−.61**	−.66**	−.65**	-	.45**	.41**	.66**	.49**	.64**	−.23	−.07	−.16*
8	2.09 (1.05)	2.47 (1.18)	−.42**	−.40**	−.43**	−.34**	−.37**	−.37**	.41**	-	.61**	.40**	.63**	.60**	−.18	−.33	−.19**
9	1.94 (.98)	2.43 (1.26)	−.33**	−.33**	−.33**	−.38**	−.40**	−.33**	.43**	.57**	-	.35**	.73**	.65**	−.21	−.32	−.20**
10	2.79 (1.22)	2.76 (1.54)	−.27**	−.30**	−.27**	−.49**	−.62**	−.54**	.61**	.31**	.38**	-	.42**	.56**	−.32	.05	−.15*
11	1.87 (.96)	2.23 (1.10)	−.39**	−.41**	−.41**	−.36**	−.33**	−.39**	.44**	.55**	.62**	.38**	-	.69**	−.19	−.33	−.17*
12	2.33 (1.15)	2.54 (1.37)	−.42**	−.40**	−.33**	−.49**	−.44**	−.47**	.55**	.48**	.48**	.41**	.52**	-	−.21	.10	−.24**
13	.32 (.25)	.40 (.26)	−.11	−.20	−.13	−.01	.08	.07	.03	.29*	.25*	−.16	.40**	.19	-	.45	−.09
14	.39 (.24)	.25 (.23)	−.01	.00	.00	−.01	.05	.05	.11	.00	−.17	.04	−.11	.03	.32**	-	.19**
15	3.94 (1.19)	4.40 (1.33)	.37**	.38**	.36**	.27**	.25**	.24**	−.22*	−.28**	−.27**	−.17**	−.27**	−.31**	−.17	−.11	-

The correlations of Study 1 can be found below the diagonal, above the diagonal are the correlations of Study 2.

Note: 1 =  enthusiastic; 2 =  cheerful; 3 =  optimistic; 4 =  contented; 5 =  calm; 6 =  relaxed; 7 =  tense; 8 =  gloomy; 9 =  depressed; 10 =  worried; 11 =  miserable; 12 =  uneasy; 13 =  intra-class correlation for negative emotions; 14 =  intra-class correlation for positive emotions; 15 =  intrinsic motivation; *p<.05; **p<.01.

#### Moderation Analysis

In a first series of models, we tested whether the different emotions were related to intrinsic motivation. To this end, we tested all specific emotions in separate multilevel regression models. The results of these analyses revealed that the specific positive emotions related significantly and in a positive way, while the specific negative emotions related significantly and in a negative way to intrinsic motivation (see Table 2). In particular, positive relationships with intrinsic motivation were found for enthusiasm (*β_1_* = .50; *p*<.01), cheerfulness (*β_1_* = .48; *p*<.01), optimism (*β_1_* = .57; *p*<.01), contentedness (*β_1_* = .41; *p*<.01), calmness (*β_1_* = .36; *p*<.01), and relaxation (*β_1_* = .33; *p*<.01), while negative relationships were found for tenseness (*β_1_* = −.24; *p*<.01), gloominess (*β_1_* = −.39; *p*<.01), depression (*β_1_* = −.44; *p*<.01), worry (*β_1_* = −.20; *p*<.01), miserableness (*β_1_* = −.44; *p*<.01), and uneasiness (*β_1_* = −.40; *p*<.01).

**Table 2:** Parameter estimates of Model 1 and Model 2 (diary study).

	Model 1		Model 2			
	*motivation_ij_ = β_0j_+β_1j_em_ij_*		*motivation_ij_ = β_0j_+β_1j_em_ij_+β_2_diff_j_+β_3_em_ij_xdiff_j_*			
	β_0_	β_1_	β_0_	β_1_	β_2_	β_3_
enthusiastic	3.88**	.50**	3.85**	.47**	.07	.70*
cheerful	3.88**	.48**	3.85**	.45**	.07	.86**
optimistic	3.88**	.57**	3.85**	.54**	.07	.60†
contented	3.89**	.41**	3.86**	.38**	.07	.81**
calm	3.89**	.36**	3.86**	.33**	.06	1.00**
relaxed	3.89**	.33**	3.86**	.28**	.07	1.16**
tense	3.88**	−.24**	3.87**	−.22**	.00	−.42*
gloomy	3.89**	−.39**	3.87**	−.38**	.00	−.43†
depressed	3.89**	−.44**	3.87**	−.35**	.00	−.86**
worried	3.89**	−.20**	3.87**	−.17*	−.00	−.55*
miserable	3.89**	−.44**	3.87**	−.36**	.00	−.87**
uneasy	3.89**	−.40**	3.87**	−.36**	−.00	−.53**

Note: motivation  =  intrinsic motivation; em  =  emotion; diff  =  intra-class correlation of positive or negative emotions; *p<.05; **p<.01, † <.10.

In the second series of models, we tested whether emotion differentiation moderated the relationship between the different emotions and intrinsic motivation. To this end, we predicted intrinsic motivation from one particular specific emotion, emotion differentiation, and the (cross-level) interaction between the specific emotion and emotion differentiation. Again, we estimated different models for the different specific emotions. The estimates of these models are shown in Table 2. In line with Hypothesis 1, we found that negative emotion differentiation negatively moderated the relation between each specific emotion and intrinsic motivation. In particular, a negative moderation effect was found for tenseness (*β_3_* = −.42; *p*<.05), depression (*β_3_* = −1.86; *p*<.01), worry (*β_3_* = −.55; *p*<.05), miserableness (*β_3_* = −.87; *p*<.01), and uneasiness (*β_3_* = −.53; *p*<.01), while the moderation effect for gloominess approached conventional levels of significance (*β_3_* = −.43; *p*<.10). Positive emotion differentiation positively moderated the relation between enthusiasm (*β_3_* = .70, *p*<.05), cheerfulness (*β_3_* = .86, *p*<.01), contentedness (*β_3_* = .81, *p*<.01), calmness (*β_3_* = 1.00, *p*<.01), and relaxation (*β_3_* = 1.16, *p*<.01) on one hand and intrinsic motivation on the other hand, while the moderation effect for optimism approached statistical significance (*β_3_* = .60, *p*<.10). Together, these findings support Hypothesis 2.

### Discussion

The results of the diary study were in line with our hypotheses. That is, emotion differentiation moderated the relationship between the specific emotions and intrinsic motivation, with the moderation effect of negative and positive emotion differentiation differing in sign. Regarding negative emotion differentiation, we observed that employees who could not easily discriminate the different negative emotions (i.e., poor negative differentiators) showed a stronger negative relationship between the different negative emotions and intrinsic motivation than employees who were able to do so (i.e., good negative differentiators). This is in line with previous research that has demonstrated that these people are worse in regulating their emotions [42]. In contrast, for good positive differentiators the positive relationship between the positive emotions cheerfulness, calmness, enthusiasm, and relaxation on one hand and intrinsic motivation on the other hand was weaker than for poor positive differentiators. As such, our findings confirmed the claim by Tugade, Feldman Barrett, and Gross [49] that being a good differentiator is not necessarily related to more positive outcomes. More specifically, our data showed that, at least with respect to motivation-related phenomena within SDT, being a poor positive differentiator might be beneficial.

We also found that, for high valence, low arousal emotions (i.e., contentedness, relaxation, and calmness), poor positive differentiators showed a strong positive relation between the experience of these emotions and intrinsic motivation. The reason is that poor positive differentiators do not only feel relaxed or calm, but at the same time they also feel optimistic, cheerful, and excited. As a result, when their feeling of, for example, relaxation increases, a mixture of positive emotions is triggered, thereby augmenting their intrinsic motivation. This finding is in line with previous SDT research on positive and negative emotions, which has shown that people who experience positive emotions are more intrinsically motivated [16], [17]. In contrast, our data also showed that the intrinsic motivation of good positive differentiators did not increase when they felt more relaxed or calm (probing the slopes for significance revealed that the slopes for contentedness, relaxation, and calmness became non-significant when people scored more than .96, .71, and .50 SD′s below the mean ICC respectively). This finding is in line with research on specific emotions, where relaxation and calmness are conceived of as positive deactivating emotions, or emotions for which the positive part relates to motivation only in the long term, while the deactivating part stimulates amotivation in the short term [66]–[68].

Although diary studies have important strengths [69], they are also subject to some limitations. First, the variables are not measured at the time when they are experienced; hence, recall biases may occur [69]. Second, all questions are answered at the same moment in time. This makes the results susceptible for common method bias [70]. To replicate the findings of our first study, and to prevent recall bias from affecting our findings, we also tested our hypotheses in an experience sampling study. Such an experience sampling design captures real-life experiences at random moments in time [69].

## Study 2

### Method

#### Participants

Thirty-four Belgian employees of different companies and governmental services (8 men and 26 women) consented to take part in this study. The mean age of the participants was 40.71 years (*SD* = 14.13). Most participants (82.4%) worked fulltime.

#### Procedure

Before the start of the study, employees signed an informed consent and answered some demographic questions. Employees participated in the experience sampling study during five consecutive working days. At four semi-random times, two before and two after noon, participants received an email or a text-message upon which they were required to rate their current emotions by means of an online questionnaire. Fifteen minutes after the first, they received a second email or a text-message that invited them to rate their intrinsic motivation. By introducing a time lag in the design (i.e., measuring emotions 15 minutes before measuring intrinsic motivation), we lowered the possibility that our findings were affected by common method bias (see [70]). Three participants responded using paper and pencil. Data that did not comply with the prescribed procedure were discarded; for example, when a participant did not complete the questions timely, or when a participant did not respect the 15 minutes time lag. After data cleaning, 197 responses (a response rate of 57.94%) remained. Note that we did not ask permission for this study to a review board (see also Study 1).

#### Measurements

All questions were selected from valid and reliable questionnaires. The original formulation of the questions was changed to measure emotions and motivation experienced at that particular moment. All items were answered on a seven-point rating scale ranging from *totally disagree* (1) to *totally agree* (7).

Emotions were assessed with the same questionnaire as in the diary study, namely the experience and evaluation of work questionnaire (QEEW; [55]).

Intrinsic motivation was assessed with all four items of the intrinsic motivation subscale from the situational motivation scale [71], which measures state intrinsic motivation. In particular, the validated Dutch translation of Bos-Nehles [72] was used in this study. Cronbach's alpha of this scale was .95. A sample item is: “At the moment I do this task because I think that this task is interesting.”

Positive and negative emotion differentiation indices were again obtained by computing the intra-class correlation coefficient (ICC) between the positive/negative emotion ratings of that individual across his/her different assessments.

### Results

#### Power analysis

Because there were fewer participants and fewer repeated measurements in the experience sampling study than in the daily diary study, we first tested whether there was sufficient statistical powerful to detect important cross-level interactions. To do so, we computed the statistical power for detecting cross-level interactions using the Monte Carlo tool of Mathieu, Aguinis, Culpepper, and Chen [73]. As input for the power calculations, we used the parameter estimates of the model with the largest (i.e., relaxation) and smallest (i.e., tension) statistically significant cross-level interactions of Study 1. The results of these power calculations showed that in Study 1 the statistical power for detecting both cross-level interactions was 1, while in Study 2 the statistical power was 1 for the largest, while it was .99 for the smallest statistically significant cross-level interaction of Study 1. In other words, to the extent that the effect sizes of the cross-level interactions are similar to those found in Study 1, the present study has sufficient statistical power to detect them.

#### Descriptive Statistics

The means, standard deviations, and correlations of all study variables are reported above the diagonal in Table 1. Note that the correlations of the specific emotions and intrinsic motivation were computed on group-mean centered data, whereas the correlations involving the intra-class correlations were computed on data aggregated to the person-level. All positive emotions (e.g., calm-relaxed: *r* = .68, *p*<.01), and all negative emotions (e.g., depressed-miserable: *r* = .73, *p*<.01) were positively correlated to each other. Positive emotions were negatively related to the negative ones (e.g., calm-tense: *r* = −.59, *p*<.05). Intrinsic motivation correlated positively with enthusiasm, (*r* = .24, *p<*.01), cheerfulness (*r* = .26, *p*<.01), calmness (*r* = .18, p<.05), and relaxation (*r* = .22, p<.01), while intrinsic motivation related negatively to all negative emotions (e.g., uneasy-intrinsic motivation: *r* = −.24, *p*<.01).

#### Moderation Analysis

The moderating effect of emotion differentiation on the relation between the specific emotions and intrinsic motivation was tested by means of a series of two-level multilevel regression models using the lme4 package in R. The analytical procedure was identical to that in Study 1.

In a first series of models, we tested whether the different emotions (whose scores were group-mean centered) were significantly related to intrinsic motivation. To this end, we entered each specific emotion separately into our model and tested whether the relationship differed across individuals. The fixed effects of these first models are shown in Table 3. In general, the different emotions predicted intrinsic motivation, with positive emotions being positively related and negative emotions being negatively related to intrinsic motivation. In particular, a positive relationship was found for enthusiasm (*β_1_* = .36; *p*<.01), cheerfulness (*β_1_* = .37; *p*<.01), optimism (*β_1_* = .14; *p*<.10), contentedness (*β_1_* = .27; *p*<.10), calmness (*β_1_* = .31; *p*<.05), and relaxation (*β_1_* = .32; *p*<.01), while we found a negative relationship for tenseness (*β_1_* = −.25; *p*<.05), gloominess (*β_1_* = −.31; *p*<.01), depression (*β_1_* = −.31; *p*<.01), worry (*β_1_* = −.22; *p*<.05), miserableness (*β_1_* = −.31; *p*<.01), and uneasiness (*β_1_* = −.34; *p*<.01).

**Table 3:** Parameter estimates of Model 1 and Model 2 (experience sampling study).

	Model 1		Model 2			
	*motivation_ij_ = β_0j_+β_1j_em_ij_*		*motivation_ij_ = β_0j_+β_1j_em_ij_+β_2_diff_j_+β_3_em_ij_xdiff_j_*			
	β_0_	β_1_	β_0_	β_1_	β_2_	β_3_
enthusiastic	4.54**	.36**	4.55**	.27**	1.66*	.78**
cheerful	4.56**	.37**	4.56**	.28**	1.69*	.68*
optimistic	4.54**	.14†	4.54**	.06	1.66*	.76*
contented	4.54**	.27†	4.55**	.21†	1.64*	1.33*
calm	4.54**	.31**	4.55**	.24*	1.68*	.58
relaxed	4.55**	.32**	4.55**	.24*	1.68*	.67
tense	4.55**	−.25**	4.51**	−.24*	.04	−.44
gloomy	4.56**	−.31**	4.53**	−.20*	.04	−.67†
depressed	4.54**	−.31**	4.51**	−.26**	.04	−.49
worried	4.53**	−.22*	4.50**	−.20*	.04	−.61
miserable	4.54**	−.31**	4.50**	−.18†	.03	−.79†
uneasy	4.55**	−.34**	4.51**	−.24**	.11	−.60†

Note: motivation  =  intrinsic motivation; em  =  emotion; diff  =  intra-class correlation of positive or negative emotions; *p<.05; **p<.01; † <.10.

In the second series of models, we tested whether emotion differentiation (which was grand-mean centered) moderated the relationship between the different emotions and intrinsic motivation. To do so, we entered emotion differentiation and the interaction between the specific emotions and emotion differentiation into the first model (slopes that were random in the previous step were specified to be random in these models as well). Table 3 shows the fixed effects of these models. For negative emotion differentiation, the moderation effect approached conventional levels of significance for gloominess (*β_4_* = −.67; *p*<.10), miserableness (*β_4_* = −.79; *p*<.10), and uneasiness (*β_4_* = −.60; *p*<.10), and was in the predicted –negative– direction. Positive emotion differentiation, in turn, positively moderated the relationship between enthusiasm (*β_4_* = .78; *p*<.01), cheerfulness (*β_4_* = .68; *p*<.05), optimism (*β_4_* = .76; *p*<.05), and contentedness (*β_4_* = 1.33; *p*<.05) and intrinsic motivation. No moderation effect was found for calmness (*β_4_* = .58; *ns*), relaxation (*β_4_* = .67; *ns*), tenseness (*β_4_* = −.44; *ns*), depression (*β_4_* = −.49; *ns*), and worry (*β_4_* = −.61; *ns*). Together, these findings provided mixed support for both hypotheses 1 and 2.

### Discussion

Although the effects in our experience sampling study were less strong than those of the daily diary study (which might be due to differences in the procedure –measuring at the end of the working day or measuring momentarily), the findings were largely in line with those of the diary study.

In particular, we have shown that for good negative differentiators, the relationship between gloominess, miserableness, and uneasiness on one hand and intrinsic motivation on the other hand is marginally weaker than for poor negative differentiators. Conversely, poor positive differentiators showed a stronger positive relationship between enthusiasm, cheerfulness, optimism, contentedness and intrinsic motivation. For example, for poor positive differentiators, intrinsic motivation was positively related with the experience of contentment, which is in line with results from recent SDT research on positive and negative emotions [15], [16]. Conversely, for good positive differentiators, intrinsic motivation was unrelated, or even negatively related to the experience of this emotion (probing the slope for significance revealed that the slope for contentment was nonsignificant for employees scoring .13 SD′s above the mean ICC). Because in research on specific emotions contentment is conceived of as positive deactivating emotions with the positive part relating to motivation in the long term, while the deactivating part stimulates amotivation in the short term, this finding is in line with the theorizing on specific emotions [66]–[68].

## General Discussion

Both the diary and the experience sampling study demonstrated that both positive and negative emotion differentiation moderated the relationship between different specific emotions and intrinsic motivation. In particular, the effect of the specific emotions on intrinsic motivation depended on whether employees experienced these specific emotions as a mixture of positive emotions or as distinct, well-separated emotions, and the same was true for negative emotions. By doing so, our findings revealed that individual differences in emotion differentiation are central in explaining the boundary conditions for the emotion-intrinsic motivation relationship by showing that individual differences in emotion differentiation can account for individual differences in the extent to which people's motivation depends on their emotions. As such, the present findings increased our understanding of the mechanism behind the emotion-intrinsic motivation relationship and added to the growing body of research on the affective antecedents of SDT.

The few studies that have focused on emotions as antecedents of motivation within the SDT framework have mainly focused on the broad categories of positive versus negative affect [15], [16]. Whereas these studies have demonstrated that general positive and negative affect do indeed predict motivation, studies in the emotion domain have convincingly shown that different specific emotions have different action readiness components, and may therefore relate in a different way to motivation [32], [74], [75]. The present paper demonstrated that the results obtained in both research streams might be linked via the concept of emotion differentiation. In particular, for poor differentiators, intrinsic motivation increased when they experienced positive emotions, whereas it decreased when they experienced negative emotions [15], [38], [39]. Because poor differentiators are unable to differentiate between different specific emotions of the same valence, the relevant information for the occurrence of intrinsic motivation is whether the emotion is positive or negative in valence. Conversely, for good differentiators, we have shown that the relationship between the specific emotions and intrinsic motivation depended on the emotion in question, with for example relaxation and calmness being weakly or unrelated to intrinsic motivation. This implies that, for people high on emotion differentiation, the differentiation of general positive and negative affect into different specific emotions leads to different predictions in terms of the person's level of intrinsic motivation.

In line with the findings of Demiralp et al. [43], we demonstrated that positive and negative emotion differentiation should be considered different constructs. Both types of emotion differentiation were only moderately correlated and had different effects on the emotion-intrinsic motivation relationship. In particular, for negative emotion differentiation, we found that being a good negative differentiator is a desirable feature (see also [42], [49]) because it weakens the relationship between negative emotions and intrinsic motivation. For positive emotion differentiation, instead, high levels appeared to be less desirable because good positive differentiators showed a weaker positive relationship between positive emotions and intrinsic motivation. As only a few studies have focused on the concept of positive emotion differentiation, our results add to the research on this rather novel concept. Moreover, our findings are generally in line with those of previous studies. For example, while Feldman Barrett, Gross, Christensen, and Benvenuto [42] found no relationship between emotion regulation and positive emotion differentiation, Tugade, Feldmans Barrett, and Gross [49] demonstrated that good positive differentiators engaged less in self-distraction and more in behavioral disengagement. This links up with our findings as they indicate that good positive differentiators –as opposed to poor positive differentiators– are more likely to give up or withdraw effort (i.e., diminish their level of motivation) when stressors interfere with their goals. Moreover, our findings do also line up with the finding of Selby et al. [21] that people high in positive emotion differentiation draw less on their momentary positive emotions than people low in positive emotion differentiation. Integrating these results with our findings, this seems to suggest that poor differentiators not only have increased levels of emotional reactivity [48], but that they also react more strongly to the emotions they experience [21]. This implies that the experience of specific positive emotions leads to more intrinsic motivation, while for negative emotions it leads to less intrinsic motivation in poor differentiators.

The results of the present study have important practical implications as well. By underscoring the role of emotions in the elicitation of intrinsic motivation at work, they reveal that organizations might affect the intrinsic motivation of their employees by influencing their positive and negative emotional experiences. This can for example be done by involving employees in decision making, giving employees recognition for their work, or increasing the level of positive feedback, as it is known that these job redesign principles all influence the affective states of employees to the better [76], [77]. Of course, it is naive to believe that such job redesign interventions would prevent the occurrence of negative emotions. Therefore, installing an open climate in which employees can get social support when they need it might help individuals to down-regulate their negative emotions, which in turn would promote intrinsic motivation. Similarly, providing mindfulness trainings to employees can help people to better regulate their emotions because mindfulness is positively related to emotion differentiation, which in turn relates to improved emotion regulation [48]. Moreover, because negative emotion differentiation is desirable for the elicitation of intrinsic motivation, clear communication and feedback can help poor negative differentiators to pinpoint the exact cause of their negative emotions, which might help employees to crystalize their appraisals, which in turn should promote increased levels of emotion differentiation. Finally, as individual differences exist in the way people experience their emotions, managers should pay attention to each employee individually. Because individual consideration is a key dimension of transformational leadership, organizations might look for transformational leaders as they inspire people and try to meet the emotional needs of each employee individually [78].

### Limitations and future research

Some limitations should be taken into account when interpreting the results of our studies. First, even with the use of time lags, we can only hint toward causality. The reason is that causal relations can only be tested when three conditions are fulfilled: (1) causes and effects should be associated, (2) causes have to precede effects, and (3) possible spurious causes should be controlled for [79]. The first condition was fulfilled in the diary study, and the first and second conditions were fulfilled in the experience sampling study. Yet, to fulfill the third condition, randomization is needed. Consequently, true causal relations can only be found with experimental designs.

Second, different questionnaires were used to measure intrinsic motivation in the diary and experience sampling study. Whereas this allowed us to demonstrate that our findings were not measure-specific, at the same time the difference in measures can partially be responsible for the small differences that were found in both studies.

Third, we only focused on intrinsic motivation. While this is probably the type of motivation that is most affect-driven [16], other types of motivation are also relevant in a work setting (e.g., controlled motivation). Future research should thus focus on other types of motivation as well.

## Conclusion

We demonstrated that specific emotions relate to intrinsic motivation, and that individual differences in the extent to which people are able to differentiate between different emotions moderated the emotion-intrinsic motivation relationship. In particular, people who were unable to distinguish between different positive emotions (i.e., poor positive differentiators) showed a stronger relationship between these specific positive emotions and intrinsic motivation, whereas the relationship between specific negative emotions and intrinsic motivation was weaker for people who were able to distinguish between different negative emotions (i.e., poor negative differentiators). By doing so, our study supported the claim that emotions and individual differences in the experience thereof are of key importance in the motivation-generative process of SDT.

## Supporting Information

S1 Data (ZIP)
